# Can Estivation Preferences Be Used to Develop Novel Management Tools against Invasive Mediterranean Snails?

**DOI:** 10.3390/insects12121118

**Published:** 2021-12-14

**Authors:** Priscillia Hanache, Thierry Thomann, Valerie Caron, Gaylord A. Desurmont

**Affiliations:** 1 CSIRO European Laboratory, 34980 Montferrier-sur-Lez, France; priscillia74klm@hotmail.fr (P.H.); thierry.thomann@csiro.au (T.T.); 2 Faculté des Sciences et Techniques, Université Jean Monnet, 42100 Saint Etienne, France; 3 CSIRO Health and Biosecurity, Black Mountain, Canberra, ACT 2601, Australia; 4 European Biological Control Laboratory USDA-ARS, 34980 Montferrier-sur-Lez, France; gdesurmont@ars-ebcl.org

**Keywords:** land snail, aggregation, *Theba pisana*, *Cernuella virgata*, *Cochlicella acuta*, *Cochlicella barbara*, Gastropoda

## Abstract

**Simple Summary:**

Terrestrial snails living in warm and dry climates had to develop strategies to survive. Several species climb on vertical supports when temperatures rise and spend the warmest months resting, typically in groups. Understanding this behavior could be useful in developing new management tools for species that are invasive. Here, we focused on four invasive snail species, and assessed their preferences for vertical supports varying in widths and heights under laboratory and field conditions. We also tested whether the presence of other snails from the same or different species affected these preferences. The snails strongly preferred wider supports in laboratory choice tests, and one species (*Theba pisana*) preferred taller supports as well. Results were similar in the field, where more snails were found on wider and taller supports 24 h after being released. The percentage of snails found in groups on a support was strongly density-dependent. The presence of other snails or their mucus did not affect the choices made. Overall, our results point towards the possibility of developing attractive supports to trap snails in the field.

**Abstract:**

Terrestrial snails that live in hot and dry climates have developed strategies to cope with high summer temperatures. Several species estivate during the warmest months of the years by resting on vertical supports, typically in groups. Understanding how snails choose their estivation sites and aggregate may lead to the development of new management tools in areas where these snails are invasive. Here, we investigated the preferences of four snail species for vertical supports varying in widths and heights under laboratory and field conditions, and tested whether the presence of conspecifics or snails of other species affected these preferences. The results show that the snails strongly preferred wider supports in laboratory dual-choice tests, and one species (*Theba* *pisana*) showed a consistent preference for taller supports as well. These results were confirmed in the field, where more snails were found on wider and taller supports 24 h after being placed in test quadrats. The percentage of snails found in groups on a support was strongly density-dependent. The presence of conspecifics or their mucus did not affect the choices of the snails, nor did the presence of snails of other species or their mucus. Taken together, these results could lead to the development of attractive supports that could be used to mass-capture snails in the field.

## 1. Introduction

Mollusks have evolved in aquatic environments, but some lineages have successfully colonized terrestrial environments [1,2]. Out of the 85,000 species of the Gastropoda (snails and slugs) species, an estimated 24,000 species are terrestrial. Land gastropods have evolved an array of morphological, physiological, ecological, and behavioral adaptations that enable them to reduce desiccation risks [3]. While some snails occupy consistently humid habitats, others have adapted to hot and dry environments, including deserts. One of the key strategies used by gastropods to cope with such harsh environments is estivation.

Estivation is a diapause occurring during summer. It is an extended resting period that is associated with a reduction in the metabolic rate, including changes to enzymes and protein activities [4,5]. In several Mediterranean snails, estivation occurs after snails have climbed onto vertical supports that are usually plants, but that can also be manmade structures. By climbing, snails avoid hot ground temperatures [6,7]. Indeed, the temperature decreases as the distance from the ground increases [8]. However, being exposed to the sun can also make their body temperature 10 °C higher than the ambient temperature [9]. In Australia, clustering snails can be found on robust weeds in the field, which may provide increased moisture [10].

Estivation behavior seems to be triggered by a lack of moisture, but not by a lack of food [11]. Some species break estivation and become active after rain during summer, which can be energy-consuming [12]. A recent study showed that, for some species, snail movement over summer increases with the relative ground humidity [13]. Resting higher from the ground would prevent them from being unnecessarily activated by rain or dew, which is more prominent closer to the ground [14].

Estivating snails are often found aggregating in large numbers. McQuaid et al. (1979) showed that *Theba pisana* (Müller) (Gastropoda: Helicidae) individuals resting in clumps on vertical supports had lower internal temperatures than isolated snails at the same height. Forming clumps may therefore benefit snails by providing them with better protection from high temperatures. It could also reduce predation risks, as seen in aggregating marine mollusks [15]. Such benefits may have led to the evolution of aggregation behavior in snails [16]. However, it is unclear whether aggregations occur because of a lack of space and competition for the same estivation sites, or whether they occur by choice. The latter possibility implies that snails either actively search for their conspecifics, or randomly encounter conspecifics and decide to begin estivation next to them. Active searching for conspecifics might be achieved via vision or olfaction.

Terrestrial gastropods have been shown to track odors, conspecifics, or prey items using the sensory receptors present on their tentacles, either by touch (tropotaxis), or by tracking the aerial plumes of chemical compounds (anemotaxis) [17]. Some species can use mucus to find other snails, but this ability is highly variable and species-specific (e.g., [18]). Anemotaxis has been shown for several species using olfactometers [19,20,21]. However, this behavior is not consistent for all species [22].

Vision could also be used by snails to orient themselves and to possibly find other individuals. The eye of terrestrial gastropods is thought to be too simple to see objects with any level of detail, but they may still detect basic shapes [23]. Zanforlin (1976) showed that the snail, *T. pisana*, prefers to orient towards larger forms, irrespective of the form shape [24]. *Littorina irrorata* (Say) (Gastropoda: Littorinidae), a snail found in marshes, was shown to prefer vertical lines, which correspond to its natural habitat, to horizontal and diagonal lines [25]. *Otala lactea* (Müller) (Gastropoda: Helicidae), a land snail from Morocco, was shown to prefer darker areas to lighter areas [26].

Mediterranean land snails have become invasive in different areas of the world. In Australia, four species of introduced snails are considered major pests in grain crops: two globular snails, *T. pisana* (Gastropoda: Helicidae) and *Cernuella virgata* (Da Costa), and two conical snails, *Cochlicella acuta* (Müller) and *Cochlicella barbara* (Linnaeus) (Gastropoda: Geomitridae) [12]. All four species climb to higher ground when temperatures start to rise in spring, including on crops, although a proportion of conical snails also aggregate beneath structures, such as rocks or the bases of plant stems [27]. Snails present on cereal stalks are harvested and cause major grain contamination issues [12]. These snails are found in very large numbers in Australia: on fence posts, there may be thousands of individuals from more than one species. Understanding how snails find each support, and what types of supports they prefer to estivate on, could lead to the discovery of novel control methods. If snails prefer specific types of vertical supports and/or chemical cues associated with the presence of conspecifics, lures could potentially be developed to mass-trap snails at the beginning of the estivation period before they climb up cereal stalks.

The aim of this study was to investigate the estivation and aggregation behaviors of four invasive snail species. Firstly, laboratory choice tests were conducted to investigate the behavioral responses of snails when in the presence of different types of vertical supports and in the presence of other snails. Secondly, laboratory findings were tested in the field in a realistic setting, at a time when snails naturally estivate. Specifically, we tested the following hypotheses:

1. Snails show preferences for supports with certain physical characteristics. Two physical characteristics were tested: the width of the support and the height of the support. This hypothesis was tested under laboratory and field conditions;
2. Snails prefer vertical supports with conspecifics already present;
3. Snails prefer vertical supports with traces of mucus from conspecifics;
4. The presence of other snail species, or the mucus of other snail species, affects the preferences of snails for vertical supports.

Most experiments were repeated twice: once in the European spring (March–May), when snails are active in the field, and once in the European summer (July), during the natural estivation period of the four snail species tested.

## 2. Materials and Methods

### 2.1. Snails

Four species of snails were used in the study: two conical (*C. acuta* and *C. barbara*) and two globular species (*C. virgata* and *T. pisana*). All four species are originally from the Mediterranean region [28] but have become invasive in Australia [12,29,30] and elsewhere (e.g., [31,32,33,34]). *C. virgata* can attain a shell diameter of 20 mm, and *T. pisana,* a shell diameter of 30 mm. The conical snails are smaller, with *C. acuta* being < 18 mm tall and *C. barbara* < 10 mm tall [35]. All four species exhibit broadly similar behaviors and ecologies. While they can cause damage to emerging crops and pastures, they also feed extensively on dead plant matter [35]. Snails lay eggs in clusters in the topsoil from late autumn to winter, feeding and growing over winter and spring. With increasing temperatures and decreasing moisture in late spring/early summer, they begin climbing to higher ground, such as on vegetation, stubble, and fence posts. Rain events during estivation can trigger activity and snails may move down from their supports for short periods of time before climbing back and resuming estivation [12,35].

### 2.2. Sampling and Rearing Conditions

For the laboratory choice tests, *C. acuta* (mean height 0.8 ± 0.1 cm) and *T. pisana* (mean diameter 0.7 ± 0.1 cm) individuals were collected in France in February 2019 (late European winter) in St-Vincent-de-Barbeyrargues (43.705833, 3.878333). *Cochlicella barbara* (mean height 0.7 ± 0.1 cm) individuals were collected in February 2019 in Montferrier-sur-Lez (43.684167, 3.875278). *Cernuella virgata* individuals were not found in the field at the time and were excluded from the spring tests.

The individuals used for the summer field tests were collected in France in May 2019 (late European spring), at the following locations: *T. pisana* in Aigues-Mortes (43.564167, 4.187778); *C. virgata*: in Lunel (43.662194, 4.136608); and *C. acuta* in St-Vincent-de-Barbeyrargues (43.705833, 3.878333). All snails were collected on vertical supports, indicating that they had started estivating. *Cochlicella barbara* had not yet started estivation at the time (i.e., they were absent from vertical supports in the field) and were excluded from the summer tests.

All individuals of the same species were stored together in a large transparent meshed plastic box with wet absorbent paper placed at the bottom. Snails were often found resting in groups on the vertical walls on their containers, exhibiting a behavior close to their estivation behavior. They were regularly offered leaves of *Sonchus oleraceus* as food. Snail colonies were maintained, and laboratory experiments were performed at a controlled photoperiod of 12:12 L:D, with LEDs at 620 lux. The ambient temperature was 21.4 ± 1 °C, and the relative humidity was 42 ± 8%.

### 2.3. Experimental Setup

#### 2.3.1. Choice Test under Laboratory Conditions

To determine if snails prefer vertical supports with certain characteristics, we used supports varying in widths and heights, in dual-choice tests in experimental arenas. The tests were conducted once in spring with *T. pisana*, *C. acuta*, and *C. barbara*, and once in summer with *T. pisana*, *C. acuta*, and *C. virgata*.

Each individual was placed in the center of a circular arena of a 24-cm diameter for *C. acuta* and *C. barbara*, and a 40-cm diameter for *T. pisana* and *C. virgata*. The arena was surrounded by white cardboard to create a white background. *Theba pisana* and *C. virgata* were restrained to a quarter of the arena by the addition of two additional white cardboard walls. The ground of the arena was made of black self-adhesive film (“Uni schwarz lack”, d-c-fix^®^, Weißbach, Germany), the substrate on which they moved the fastest in the preliminary tests. The vertical supports were black, as the preliminary tests indicated that black supports with a white background yielded the best results.

To test the preferences for supports of different widths, each snail tested had the choice between two vertical black paper strips of equal height (10.5 cm) and with different widths (three widths tested: 0.4 cm, 1.6 cm, and 6.4 cm) placed on the white background, with 9 cm between the two supports. Six combinations were tested: 0.4 vs. 1.6; 0.4 vs. 6.4; 1.6 vs. 6.4; 0.4 vs. Nothing; 1.6 vs. Nothing; 6.4 vs. Nothing. The “Nothing” treatment was a white rectangle in the background, located where a band would have been. The snail was placed facing the space between the two strips tested. A test consisted of measuring the preference of 10 individual snails in succession, in the same arena and with the same supports, and each test was repeated five times for each combination tested (10 snails tested = 1 replication; 5 replications per combination), and replications were done during five consecutive days. To avoid biases, the location of the bands in the arena (left or right) was changed between replications. No snail was used twice the same day, and the ground of the arena was thoroughly cleaned with water after each snail was tested in order to avoid potential mucus-related biases. To avoid biases related to the timing of the tests, the different replications of the choice tests were distributed at different times of the day. The arenas were lit vertically to avoid shadows inside the arenas.

The preferences for supports of different heights were tested following the same protocol. For these tests, the width of the strips was always 1.6 cm. Two heights were tested: 2.5 cm and 10.5 cm. There were three combinations tested: 10.5 vs. 2.5; 10.5 vs. Nothing; and 2.5 vs. Nothing.

It was considered that a snail had made a choice if it made contact with the support and started climbing or if it made contact with the blank rectangle, in the case of the “Nothing” treatment. If the snail touched the background first, or if no choice had been made after 10 min for *T. pisana* and *C. virgata,* or after 15 min for *C. acuta* and *C. barbara*, the test was ended and recorded as “No choice”. The test durations were based on the preliminary tests that showed that *T. pisana* and *C. virgata* were more active in the arena setting than the other two species.

Graphical illustrations of the different experimental setups are available as Supplementary Material (Figure S1a).

#### 2.3.2. Attraction to Conspecifics

To test if supports with conspecifics were more attractive to snails than supports without them, choice tests were set up in the laboratory as follows: The snails had the choice between two supports in the same arena. For these tests, the supports were made of two black plastic water pipes (HDPE) (10.5 cm in height and 2.5 cm in diameter). One support had 10 snails of the same species resting on it, and the other had no snails on it. Similar to the other choice tests, the snails tested were placed in the center of the arena, at the same distance from the two poles, which were placed 1 cm from the edge of the arena (Figure S1b). A total of 10 snails were tested per replication, and 5 replications were done for each snail species tested. As in previous tests, the position of the tubes (left or right) was changed between each replication; *T. pisana* and *C. virgata* were left in the arena for 10 min; and *C. acuta* and *C. barbara* were left in the arena for 15 min.

#### 2.3.3. Attraction to Mucus

To test if snails are attracted by the mucus of their conspecifics, choice tests were set up in the laboratory with the following procedure: For each test, a “guide” snail and a “test” snail were used. First, the guide snail was placed in the arena with a single pole of a 10.5-cm height and a 2.5-cm diameter facing it. Once the guide snail had reached the pole, the snail was removed from the arena. A second pole was then placed 9 cm from the first pole, and at an equal distance from the center, then the test snail was placed in the center of the arena facing the two poles (Figure S1c). We then noted whether the test snail chose a pole or did not make a choice. This test was repeated for 50 pairs of snails (10 test snails per replication, and 5 replications) for each species, and the side where the second pole was placed was changed between replications.

#### 2.3.4. Attraction to Another Species

To test if the presence of snails of other species affects snail choice, we used *T. pisana* and *C. acuta*. The two experiments described above (conspecific attraction and mucus attraction) were repeated by mixing species. Specifically, *T. pisana* had the choice between a support with 10 *C. acuta* snails and an empty support, and vice versa. For the mucus attraction experiment, *T. pisana* had the choice between a pole with mucus traces of a *C. acuta* “guide” snail, and a pole without traces of mucus, and vice versa.

#### 2.3.5. Choice Test under Field Conditions

To complement the results from the laboratory, a field trial was conducted with poles of different heights and widths, in six quadrats of 1 m^2^ in a grass field located near the laboratory. Each quadrat had nine poles made of PVC pipes covered with black self-adhesive film (“Uni schwarz lack”, d-c-fix^®^, Weißbach, Germany), lined up in three rows. In three of the quadrats, the diameter of the poles varied (3, 7.5, and 10.5 cm) with a constant height of 80 cm, and, in the other three, the height varied (10, 80, and 160 cm) for a constant diameter of 10.5 cm (Figure S1d). The grass inside the quadrats was cut prior to the experiments. There was no barrier to confine the snails to the quadrats. Each field test was conducted as follows: a total of 100 snails of the same species were placed in a quadrat in the late afternoon. They were placed homogeneously in the quadrat so that approximately the same number of snails would be close to each pole at the beginning of the test. They were lightly sprayed with water so that they would become active and move more easily. The number of snails that climbed on each pole within the quadrat was counted the following day (approx. 20 h after starting the test). Single snails and aggregated snails (i.e., snails in groups of two or more with their shells touching) were counted separately. We removed all the snails that did not climb and that were still on the ground, and we cleaned the poles with water to remove traces of mucus between tests. Nine replicates per snail species (two species tested: *C. virgata* and *T. pisana*) and the type of quadrat (height or width) were carried out.

### 2.4. Statistical Analyses

The results of the laboratory dual-choice tests were analyzed using exact binomial tests, with the null hypothesis that the distribution between the two choices offered to the snails should be equal (α = 0.05). Snails that did not make a choice were excluded from the analyses. For each test, the percentage participation was calculated as the number of snails that made a choice divided by the total number of snails tested, multiplied per 100. The effects of the snail species and the support widths on the percentage participation was analyzed using a two-way ANOVA, with the percentage participation as the dependent variable, and with the snail species and the width of the wider support as the independent variables. The percentage participation data were arcsine-transformed to meet the assumptions of the model. A post hoc Tukey test was used to compare the means of the variables included in the model (α = 0.05).

The results of the field data were analyzed in three ways. Firstly, the association between the number of snails that climbed on a support and the percentage of snails that were found aggregated was tested using a regression analysis. To avoid potential biases due to the characteristics of the support (width, height) that varied within the quadrats, only the “medium” supports (medium heights and medium widths) were included in this analysis, as there were exactly three supports with these characteristics in every quadrat. A linear fit and a logarithmic fit of the data were both tested, and a logarithmic fit was the best fit for the data. Secondly, the effects of the support widths and heights on the numbers of snails that climbed on the supports were compared using one-way ANOVAs after a square root transformation of the data to meet the assumptions of the models, and the means were compared using a post hoc Tukey test (α = 0.05). The replicate number was added to the model as a block factor. Thirdly, the hypothesis that the differences in the numbers of snails found on the different supports in the field were proportional to the differences in the circumferences of the supports was tested using likelihood ratio analyses. For these analyses, the expected frequencies of the snail distributions were calculated on the basis of the differences in the circumferences between the supports within a quadrat. *p*-values < 0.05 reject the hypothesis that the observed frequency corresponds to the expected frequency.

All statistical procedures were performed using the statistical software, JMP15 (SAS Institute Inc., Cary, NC, USA).

## 3. Results

### 3.1. Laboratory Choice Tests

#### 3.1.1. Width of Support

**Figure 1:**
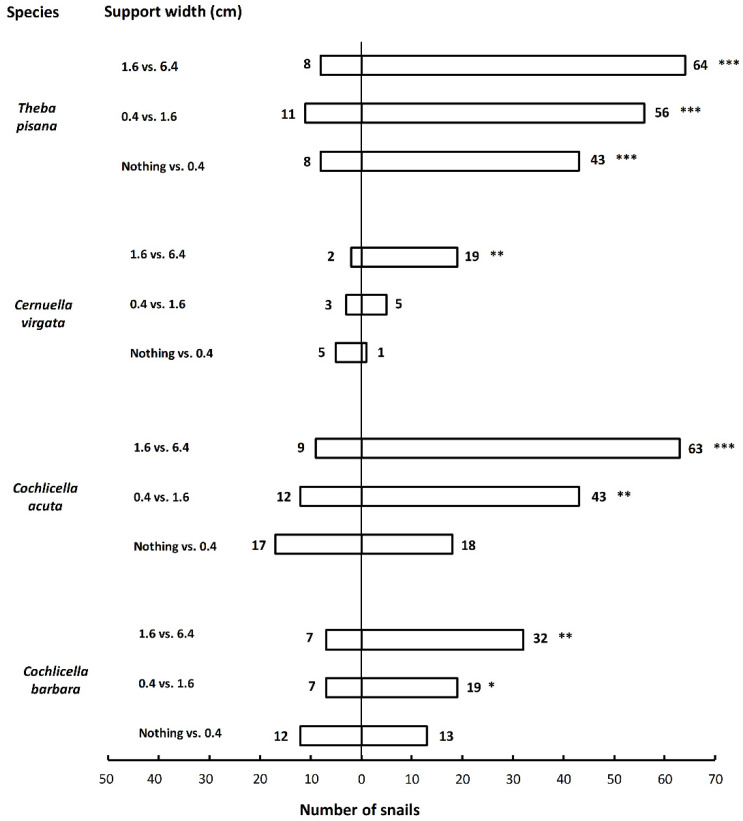
Preferences of four snail species for supports of different widths in two-choice tests under laboratory conditions. Numbers indicate the total number of snails that chose a treatment (*n* = 50 or 100 snails tested per combination). Snails that did not make a choice are not shown. Results of spring and summer tests are summed up. Asterisks indicate significant preference for a treatment (chi-square tests; *: *p* < 0.05; **: *p* < 0.001; ***: *p* < 0.0001).

**Figure 2:**
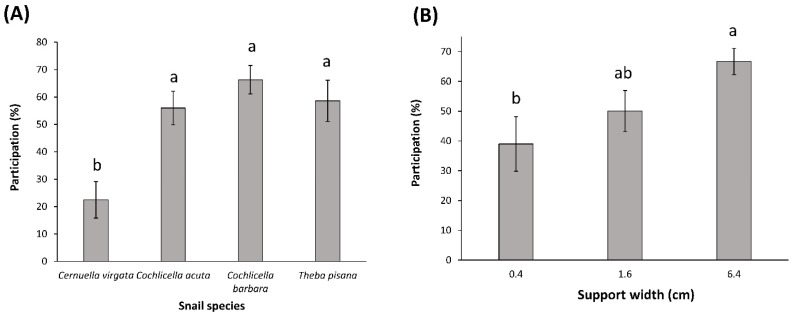
Effects of (**A**) Snail species, and (**B**) Support width (=width of the wider support tested in a two-choice test, cm) on snail participation (%, mean ± SE) in choice tests under laboratory conditions. For each figure, means with a different letter are statistically different (α = 0.05).

The four species tested showed highly consistent preferences for wider vertical supports (Table 1, Figure 1). Out of 30 tests (50 snails used per test) with supports of different widths, 22 tests showed a significant preference for the wider support, and 8 tests did not show any preference. In all 11 tests where the 6.4-cm support was present, the snails significantly preferred that support (Table 1). The four species did not show the same level of discrimination. *Theba pisana* always preferred the wider support, no matter the widths tested, while *C. barbara* and *C. acuta* preferred supports of 6.4 cm compared to 1.6 cm, and 1.6 cm compared to 0.4 cm, but did not discriminate between a 0.4-cm support and nothing. On the other hand, *C. virgata* preferred supports of 6.4 cm compared to 1.6 cm but did not discriminate between 1.6-cm and 0.4-cm supports, nor between a 0.4-cm support and nothing (Figure 1). The overall average participation (percentage of snails that made a choice) across choice tests was 54.5 ± 4.1 and varied from 12 to 94%. The snail species and the width of the wider support tested affected the snail participation (R^2^ = 0.40, F5, 24 = 4.96, *p* = 0.0029). *Cochlicella barbara*, *T. pisana*, and *C. acuta* had higher participation than *C. virgata* (F3, 24 = 4.67, *p* = 0.01) (Figure 2A). There was also higher participation when the wider support tested was 6.4 cm, compared to when it was 0.4 cm. The participation was intermediate when the 1.6-cm support was used as the wider support (F2, 24 = 3.95, *p* = 0.03) (Figure 2B).

#### 3.1.2. Height of Support

The support height affected the choices of two snail species: both *T. pisana* and *C. acuta* preferred a tall support (10.5 cm) compared to a short support (2.5 cm). However, this preference was only significant for *C. acuta* during the spring tests (χ^2^ = 3.86, *p* = 0.049), or when combining the spring and summer tests (χ^2^ = 6.48, *p* = 0.01) (Table 1). The other two species, *C. barbara* and *C. virgata*, did not discriminate between tall and short supports. None of the species showed a preference when given the choice between a short support and nothing.

#### 3.1.3. Attraction to Conspecifics

None of the snail species showed a significant preference when given the choice between a support with 10 conspecifics already present and an empty support (Table 1). In cross-species-choice tests, *T. pisana* snails did not show a preference when given the choice between a support with *C. acuta* snails and an empty support, and *C. acuta* snails did not show a preference when given a choice between a support with *T. pisana* snails and an empty support (Table 1).

#### 3.1.4. Attraction to Mucus

None of the snail species tested showed a significant preference when given the choice between a support with a mucus trace from a conspecific leading to it and a clean support (Table 1). In cross-species-choice tests, *T. pisana* snails did not show a significant preference when given the choice between a support with a trace of *C. acuta* mucus leading to it and a clean support, and *C. acuta* snails did not show a preference when given the choice between a support with a trace of *T. pisana* leading to it and a clean support (Table 1).

### 3.2. Field Choice Tests

#### 3.2.1. Aggregation Behavior

**Figure 3:**
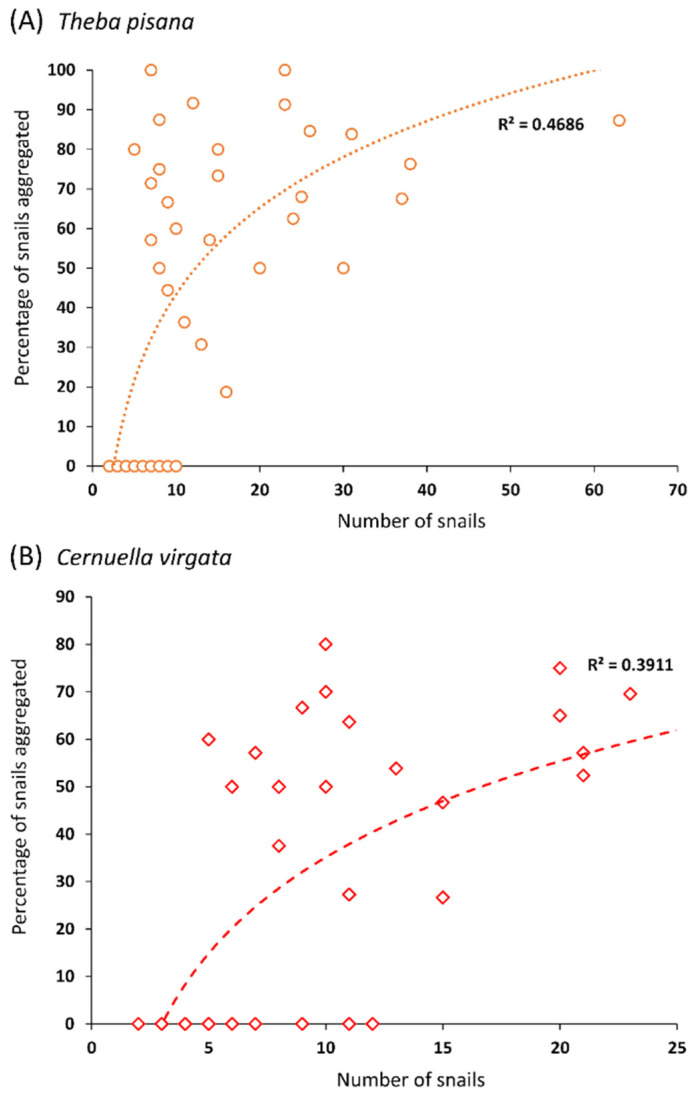
Correlation between the number of snails that climbed on a vertical support and the % of these snails that were found aggregated for (**A**) *Theba pisana*, and (**B**) *Cernuella virgata*. Dotted lines represent the logarithmic fit of the data.

A large proportion of the snails placed in the quadrats were found resting on the vertical supports the following day. For *T. pisana*, 85.3 ± 4.0 (%, mean ± SE) of the snails were found on the supports for the height choice test (min: 57; max: 97), and 82.7 ± 4.2 for the width choice test (min: 58; max: 99). For *C. virgata*, 67.9 ± 3.7 of the snails were found on the supports for the height-choice test (min: 50; max: 83), and 70.2 ± 3.9 for the width-choice test (min: 57; max: 91). A substantial number of the snails that climbed on vertical supports were found aggregated in groups of two or more. For *T. pisana*, 49.5% of the total number of snails that climbed on vertical supports across all tests were found aggregated (748 out of 1512 snails). For *C. virgata*, 36.0% of the snails that climbed on vertical supports across all tests were found aggregated (448 out of 1243). Interestingly, the percentage of snails aggregated was positively correlated with the number of snails per support for both species (*T. pisana*: y = −19.1 + 27.5 × log(x), F1,50 = 46.0 *p* < 0.0001, R^2^ = 0.47; *C. virgata*: y = −32.2 + 29.2 × log(x), F1,51 = 32.8, *p* < 0.0001, R^2^ = 0.33) (Figure 3).

#### 3.2.2. Effects of Support Widths and Heights

**Figure 4:**
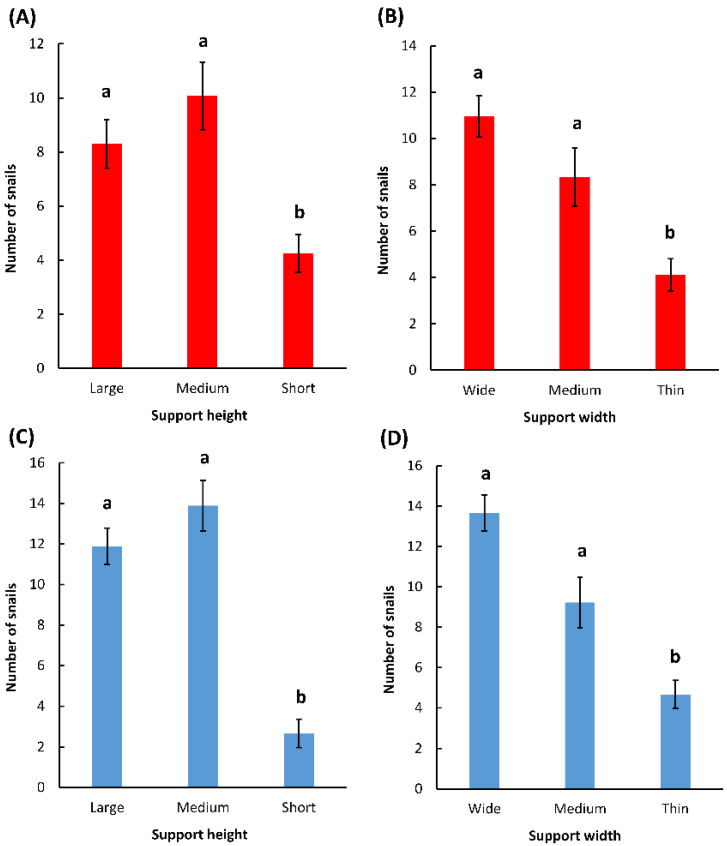
Field preferences of *C. virgata* (red) and *T. pisana* (blue) in quadrat-choice tests. (**A**) Effect of support height on *C. virgata*; (**B**) Effect of support width on *C. virgata*; (**C**) Effect of support height on *T. pisana*; (**D**) Effect of support width on *T. pisana*. For each figure, means with a different letter are statistically different (one-way ANOVA, Tukey post hoc test).

The support heights and widths affected the numbers of *T. pisana* and *C. virgata* that were found on the supports: more snails were found on large and medium supports than on shorts supports (*T. pisana*: F2, 78 = 20.4, *p* < 0.0001; *C. virgata*: F2,78 = 12.9, *p* < 0.0001), and more snails were found on wide and medium supports than on thin supports (*T. pisana*: F2, 78 = 7.4, *p* = 0.001; *C. virgata*: F2, 78 = 14.6, *p* < 0.0001) (Figure 4).

#### 3.2.3. Hypotheses Regarding the Dispersion of Snails in Field Quadrats

The hypothesis that the number of snails found on different supports is proportional to the circumferences of the supports was rejected (likelihood ratio analyses, *p*-values < 0.05) for both *T. pisana* and *C. virgata* in the support height choice tests. In these tests, all supports had the same circumference and the snails should have been equally distributed among the supports to support the hypothesis, which was not the case (Table 2). Inversely, this hypothesis was supported for both species in the width-choice tests (Table 2).

## 4. Discussion

Estivation is a critical component of the ecology of terrestrial gastropods adapted to hot and dry climates. The four snail species included in this study are known to climb on vertical supports and to form aggregations in order to spend the warmest months of the year in a resting state [3,12]. Even though this behavior is well-documented in the field, the processes of how snails choose their aestivation sites, and how they aggregate, are still poorly known. Our study aimed to fill this knowledge gap in snail ecology and to uncover behavioral preferences that could be used to develop new trapping tools for pest management. The results show a very clear pattern of preference for wider vertical supports, which was consistent in the laboratory and in the field. The presence of other snails (conspecifics or snails from other species) or their mucus did not seem to affect snail choices for vertical supports, even though they were shown to aggregate with conspecifics once they had climbed on a support in the field.

Dual-choice tests under laboratory conditions clearly showed that snails can distinguish between supports of different heights and widths. A preference for wider supports was remarkably consistent for all species. This result seems to indicate that snail vision is good enough to distinguish certain shapes from a distance. *Theba pisana* was the most sensitive species, showing preferences for the wider support in the smallest (0.4 cm vs. nothing) choice test. *Cochlicella barbara* and *C. acuta* started to show preferences towards the 1.6-cm support, and *C. virgata* only showed preferences for the 6.4-cm support. Preferences for supports of different heights were less pronounced, with *T. pisana* being the only species showing a strong preference for the tall support (10.5 cm), and *C. acuta* showing a weak (inconsistent between spring and summer tests) attraction to the tall supports.

The participation of the snails in the choice tests was shown to depend on the snail species and the widths of the supports tested: *C. virgata* was less responsive than the other snail species, and participation gradually increased with the widths of the supports tested. This latter result is consistent with the idea that wider supports elicit a stronger behavioral response in snails.

The choice tests related to the presence of conspecifics, or snails of other species, did not reveal any pattern of attraction or deterrence. The presence of snails on supports did not affect snail choice, which implies that the snails either were not able to detect the presence of other snails from a distance, or that the presence of snails did not constitute a stimulus. Similarly, the presence of mucus did not affect snail choices, in contrast to other species that have been shown to follow mucus trails (e.g., [16,36]). This lack of response to the mucus of snails of another species is somewhat surprising, as a recent study showed that the presence of traces of *T. pisana* (mucus + faeces) negatively affected the survival of *C. virgata* under laboratory conditions [37].

Taken together, the results of the laboratory choice tests strongly suggest that snails mainly use visual cues to choose vertical supports.

The general consensus regarding the vision of terrestrial gastropods is that they have poor vision, with eyes too underdeveloped to see objects in high resolution, and that snail eyes have evolved primarily to detect changes in light intensity [38]. Terrestrial snails have been commonly shown to exhibit negative phototaxis (moving away from light) [23]. This behavior may provide a mechanism for explaining snail choices in the laboratory. If snails were indeed trying to move away from the light, it makes sense that they were, in general, attracted by wider and taller black supports. Snails may not have actively looked for supports presenting certain characteristics, but, rather, may have oriented themselves toward the darkest side of the arena. It should be noted that, during the preliminary choice tests using white supports with a black background, *T. pisana* ended up more on the black walls of the arena than on the supports (data not shown), a result consistent with Zanforlin’s findings (1976). It should also be noted that the snails exhibited climbing behavior in order to rest in their rearing containers prior to and after the tests. It is therefore likely that, while they may have moved following phototaxis, they may have also been looking for a support to rest. In the early stage of estivation, snails adjust their position until a suitable place is found [3].

Snail preferences in the laboratory were, overall, very consistent between the spring and summer tests, which seems to indicate that support preferences do not depend on the phenological status of the snail (active vs. ready to estivate). However, the choice tests in the laboratory were conducted at constant temperature and humidity, which is not like the environmental context leading to snail estivation in the field. Therefore, field tests were essential in order to validate the results obtained in the laboratory.

In the field, the estivation behavior of both *T. pisana* and *C. virgata* was clearly observable. More than 80% of the snails tested were found resting on vertical supports the day after being placed in the quadrats. Interestingly, the percentage of snails forming aggregations (i.e., groups of a least two snails) increased with the number of snails found on a support. For *T. pisana*, almost all snails were found aggregated on the supports with the highest numbers of snails. This result is highly unlikely to have occurred because snails were running out of space on the supports. Indeed, the maximum space that the snails occupied on a support (63 *T. pisana* individuals) only represented 2.2% of the total surface of the support (given a surface of 1 cm^2^ per snail). Therefore, clustering is more likely to be the result of the active choice of snails to rest near conspecifics.

More snails were found in the field on wider and taller supports. These results corroborate the results found in the laboratory and may indicate that snails respond to the same stimuli in the laboratory and in the field. It is also possible that wider supports were preferred simply because they were taking up more space in the quadrats and were encountered more often by snails. Indeed, the distribution of snails on the supports of different widths matched their expected distribution if they moved randomly and climbed on the first support they encountered (Table 2). However, following the same logic, the snails should have been equally distributed among supports of different heights that had the same circumference. This was not the case, and there were consistently lower numbers of snails on the short supports (Figure 4). It is possible that short supports were not perceived as high-quality resting sites and that they were avoided by the snails.

To further explore the estivation preferences of snails, the effects of other types of support physical characteristics should be investigated (e.g., color, material used, presence of decoy snails, or cavities in the support). It also remains to be explored whether chemical cues, such as food odors, could be used to enhance the attractiveness of the support. Some snail species have been shown to respond to food odors under laboratory conditions [39,40], but investigations with *T. pisana*, *C. virgata*, and *C. acuta* revealed that they did not show strong preferences toward such odors [22]. It is also important to keep in mind that the field choice tests were 24 h long in our study, and longer-term experiments would be valuable. Indeed terrestrial snails have been shown to be able to learn and shape their foraging behavior according to past experiences [17]. The hypothesis that they may be able to assess the quality of the vertical supports present on their territory over extended periods of time, and that they make estivation choices accordingly, is worth exploring. Finally, some of the experiments conducted in the present study should ideally be repeated with invasive populations of the four snail species tested in order to make sure that their behavioral preferences are consistent with native populations.

In conclusion, this study shows that consistent patterns of preferences for certain vertical supports exist in snails that climb to estivate and provides experimental settings to further explore this aspect of snail behavioral ecology. The fact that higher numbers of snails were consistently found on wider supports in the laboratory and in the field, and on taller supports in the field, is encouraging and supports the notion that optimal supports could be developed to trap snails at the beginning of the estivation period in the field. For example, supports could be placed at regular intervals along the borders of the fields to protect them before the beginning of the estivation period, and could be removed from the field once colonized by snails. The snails removed would be disposed of in order to reduce the field populations. More importantly, the snails on supports would not be estivating on crops and would not be harvested with the grain, which is the main economic issue. The practicality of this approach needs to be evaluated, and cost/benefit ratio analyses need be performed to assess under which conditions the benefit of reducing snail numbers outweighs the costs of integrating this management method for growers. More engineered products trapping snails permanently, or controlling them directly on the support, could also be developed. The fact that all four snail species exhibited similar patterns of preferences is also promising from the perspective of pest management, as it suggests that new management tools based on estivation preferences would equally work against all four species.

## Figures and Tables

**Table 1:** Summary of the laboratory choice tests with the four snail species tested. A total of 50 snails were used per test. Chi-square tests with a *p*-value < 0.05 indicate a significant preference for one of the treatments (in bold). %part. = % participation.

			*Cochlicella acuta*				*Cochlicella barbara*				*Theba pisana*				*Cernuella virgata*			
Test Category	Season	Test	% of Choice (n)	χ²	*p*-Value	% Part.	% of Choice (n)	χ²	*p*-Value	% Part.	% of Choice (n)	χ²	*p*-Value	% Part.	% of Choice (n)	χ²	*p*-Value	% Part.
Width of support (cm)	spring	0.4 vs. 1.6	26.5 (9) vs. 73.5 (25)	**7.5**	**0.006**	68	26.9 (7) vs. 73.1 (19)	**5.4**	**0.02**	52	20.0 (4) vs. 80.0 (16)	**7.2**	**0.007**	40	X			X
	summer		14.3 (3) vs. 85.7 (18)	**10.7**	**0.001**	42	X			X	14.9 (7) vs. 85.1 (40)	**23.2**	**<0.0001**	94	37.5 (3) vs. 62.5 (5)	0.5	0.47	16
	spring	0.4 vs. 6.4	19.5 (8) vs. 80.5 (33)	**15.2**	**<0.0001**	82	12.1 (4) vs. 87.9 (29)	**18.9**	**<0.0001**	66	6.5 (2) vs. 93.5 (29)	**23.5**	**<0.0001**	62	X			X
	spring	1.6 vs. 6.4	10.5 (4) vs. 89.5 (34)	**23.7**	**<0.0001**	76	17.9 (7) vs. 82.1 (32)	**16.0**	**<0.0001**	78	17.2 (5) vs. 82.7 (24)	**12.4**	**0.0004**	38	X			X
	summer		14.7 (5) vs. 85.3 (29)	**16.9**	**<0.0001**	68	X			X	7.0 (3) vs. 93.0 40)	**31.8**	**<0.0001**	86	9.5 (2) vs. 90.5 (19)	**13.8**	**0.0002**	42
	spring	0.4 vs. nothing	42.6 (9) vs. 57.1 (12)	0.4	0.51	42	52.0 (13) vs. 48.0 (12)	<0.1	0.84	50	84.6 (11) vs. 15.4 (2)	**6.23**	**0.01**	26	X			X
	summer		64.3 (9) vs. 35.7 (5)	1.1	0.28	28	X			X	84.2 (32) vs. 15.8 (6)	**17.78**	**<0.0001**	76	16.7 (1) vs. 83.3 (5)	2.7	0.1	12
	spring	1.6 vs. nothing	73.1 (19) vs. 26.9 (7)	**5.5**	**0.02**	52	66.7 (18) vs. 33.3 (9)	3.0	0.08	74	88.2 (15) vs. 11.8 (2)	**9.9**	**0.002**	34	X			X
	summer		68.8 (11) vs. 31.2 (5)	2.2	0.13	32	X			X	89.5 (34) vs. 10.5 (4)	**23.7**	**<0.0001**	76	50.0 (5) vs. 50.0 (5)	<0.1	0.99	20
	spring	6.4 vs. nothing	91.4 (32) vs. 8.6 (3)	**24.0**	**<0.0001**	70	87.2 (34) vs. 12.8 (5)	**21.6**	**<0.0001**	78	96.3 (26) vs. 3.7 (1)	**23.1**	**<0.0001**	54	X			X
Height of support (cm)	spring	10.5 vs. 2.5	75.0 15) vs. 25.0 (6)	**3.9**	**0.049**	48	37.5 (9) vs. 62.5 (15)	1.5	0.22	48	80.0 (20) vs. 20.0 (5)	**9.0**	**0.003**	50	X			X
	summer		65.5 (19) vs. 34.5 (10)	2.0	0.09	58	X			X	97.0 (32) vs. 3.0 (1)	**29.1**	**<0.0001**	66	40.0 (2) vs. 60.0 (3)	0.2	0.65	10
	spring	2.5 vs. nothing	60.0 (12) vs. 40.0 (8)	0.8	0.37	40	68.8 (11) vs. 31.2 (5)	2.2	0.13	32	73.3 (11) vs. 26.7 (4)	3.3	0.07	30	X			X
	summer		64.3 (9) vs. 35.7 (5)	1.1	0.28	28	X			X	66.7 (14) vs. 33.3 (7)	2.3	0.12	42	40.0 (4) vs. 60.0 (6)	0.4	0.53	20
Attraction by others	spring	Congeners vs. nothing	44.1 (15) vs. 55.9 (19)	0.47	0.49	68	46.7 (21) vs. 53.3 (24)	0.2	0.65	90	44.4 (12) vs. 55.6 (15)	0.3	0.56	54	X			X
	spring	Other species vs. nothing	* 45.5 (15) vs. 54.5 (18)	0.3	0.6	66	X			X	** 42.4 (14) vs. 57.6 (19)	0.8	0.38	66	X			X
	summer	Congeners vs. nothing	45.5 (20) vs. 54.5 (24)	0.4	0.54	88	X			X	47.9 (23) vs. 52.1 (25)	0.1	0.77	96	57.1 (16) vs. 42.9 (12)	0.57	0.45	56
	summer	Other species vs. nothing	X			X	X			X	X			X	X			X
Attraction by mucus	spring	Mucus of congener vs. nothing	59.1 (39) vs. 40.9 (27)	2.2	0.14	72	57.9 (22) vs. 42.1 (16)	0.9	0.33	76	51.7 (15) vs. 48.3 (14)	<0.1	0.85	58	X			X
	spring	Mucus other species vs. nothing	* 53.3 (16) vs. 46.7 (14)	0.13	0.71	60	X			X	** 51.1 (23) vs. 48.8 (22)	<0.1	0.88	90	X			X
	summer	Mucus of congener vs. nothing	46.5 (20) vs. 53.5 (23)	0.21	0.65	86	X			X	58.3 (28) vs. 41.7 (20)	1.33	0.24	96	40.0 (12) vs. 60.0 (18)	1.2	0.27	60
	summer	Mucus other species vs. nothing	X			X	X			X	X			X	X			X

* Other species = *Theba pisana*, ** other species = *Cochlicella acuta*.

**Table 2:** Test of the hypothesis that snail distribution on supports in the field matches the differences in circumferences (widths) between the supports. Likelihood ratio analysis: *p*-values < 0.05 reject the hypothesis that the observed frequency corresponds to the expected frequency.

Species	Choice-test	Support	Height	Diameter	Observed Frequency	Expected Frequency	Likelihood Ratio	
							Chi-Square	*p*-Value
*Theba pisana*	Width	Thin	80	3	0.17 (126)	0.14	4.5	0.1
		Medium	80	7.5	0.33 (249)	0.35		
		Wide	80	10.5	0.5 (369)	0.5		
	Height	Small	10	10.5	0.09 (72)	0.33	**24.8**	**<0.0001**
		Medium	80	10.5	0.49 (375)	0.33		
		Tall	160	10.5	0.42 (321)	0.33		
*Cochlicella acuta*	Width	Thin	80	3	0.18 (111)	0.14	5.72	0.06
		Medium	80	7.5	0.36 (225)	0.35		
		Wide	80	10.5	0.47 (296)	0.5		
	Height	Small	10	10.5	0.19 (115)	0.33	**68.6**	**<0.0001**
		Medium	80	10.5	0.44 (272)	0.33		
		Tall	160	10.5	0.37 (224)	0.33		

## Data Availability

The data presented in this study are available on request from the corresponding author.

## Supplementary Materials

The following are available online at https://www.mdpi.com/article/10.3390/insects12121118/s1, Figure S1: Experimental setups.
