# Supplementary material for: Can Estivation Preferences Be Used to Develop Novel Management Tools against Invasive Mediterranean Snails?
Source: Insects. 2021 Dec 14;12(12):1118. doi: 10.3390/insects12121118 (PMC8708318; doi:10.3390/insects12121118)
Supplement: Supplementary file 1 [file insects-12-01118-s001.zip › insects-1414430-supplementary.pdf]

Can aestivation preferences be used to develop novel management tools against invasive Mediterranean snails?

Supplementary Material

A) Choice tests under laboratory conditions

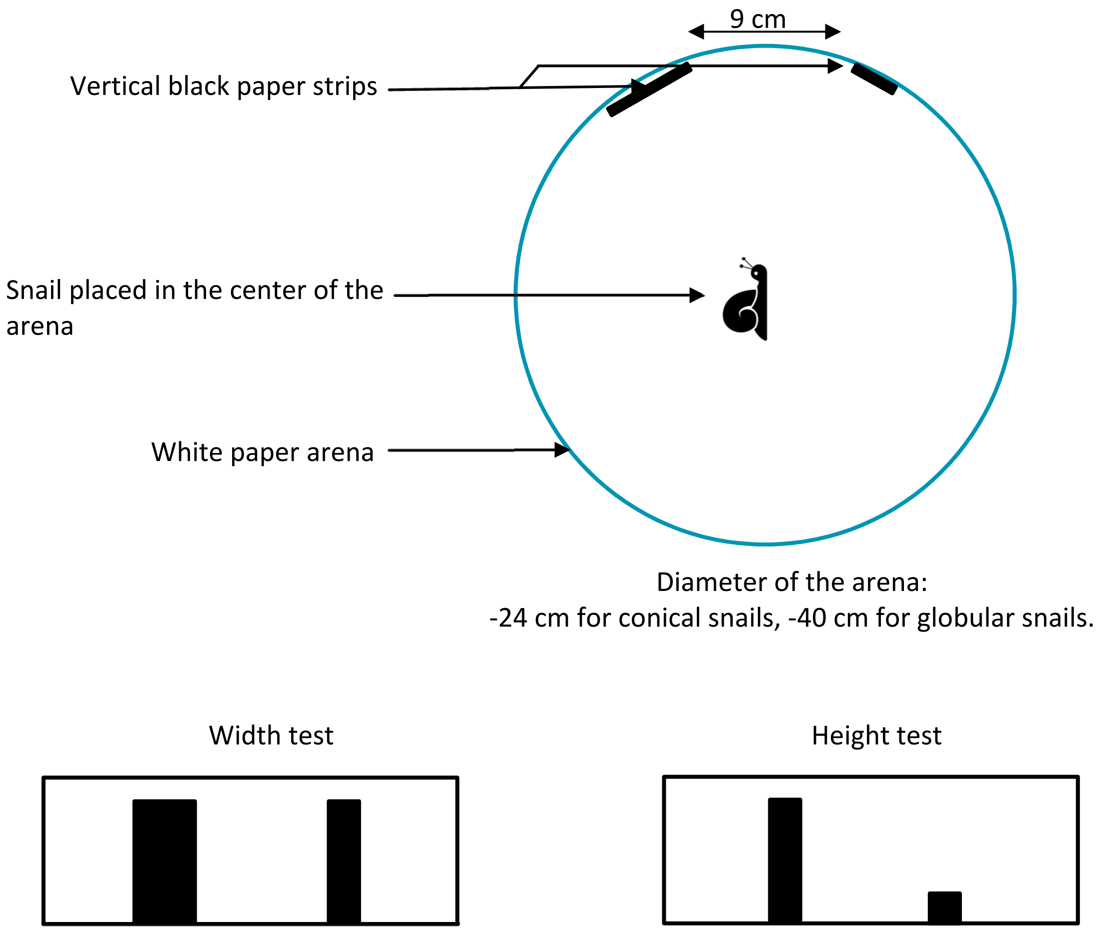

B) Attraction to conspecifics

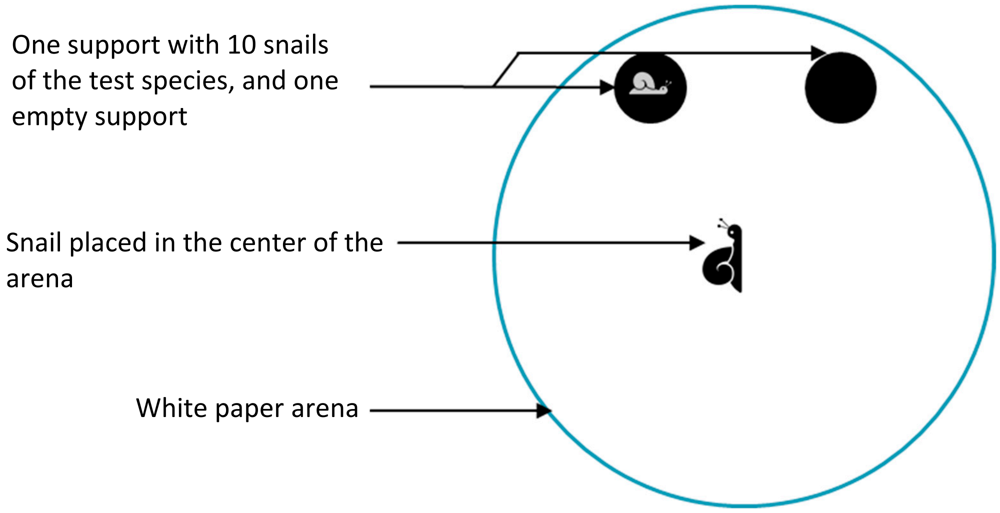

Diameter of the arena:
-24 cm for conical snails, 40 cm for globular snails.

C) Attraction to mucus

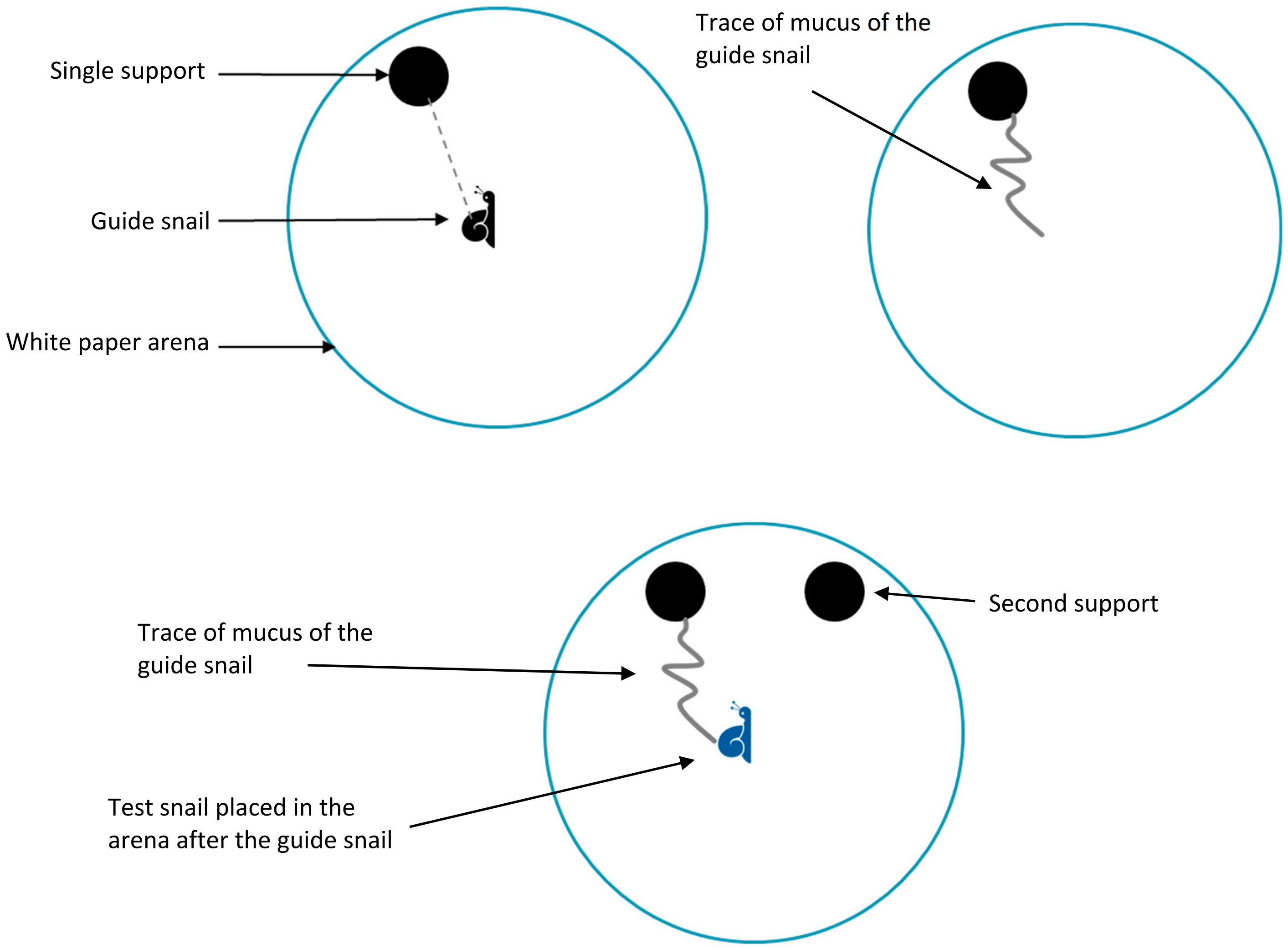

Diameter of the arena:
-24 cm for conical snails, 40 cm for globular snails.

D) Choice test under field conditions

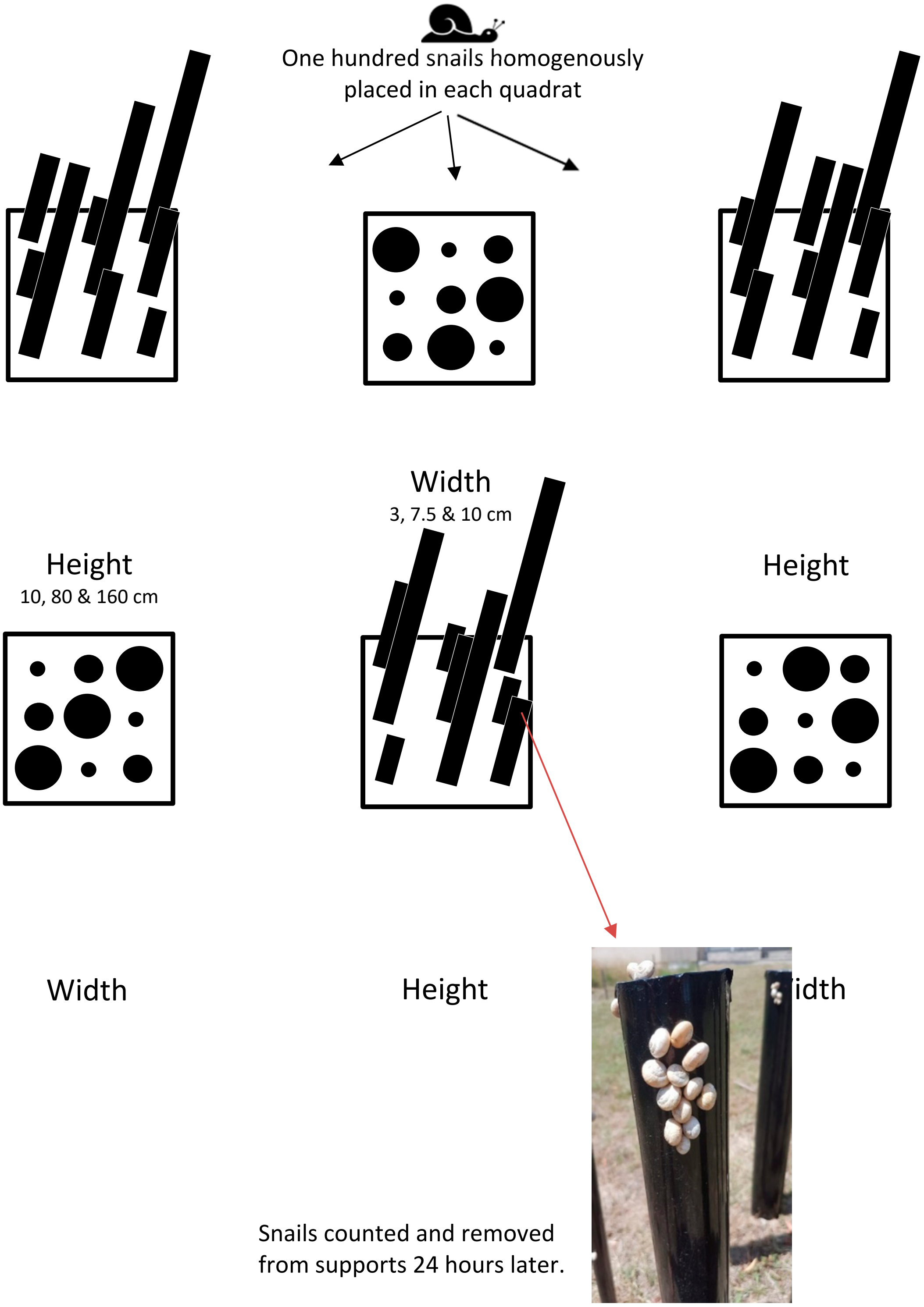

Figure S1. Experimental set ups.
